# Supportive care needs and health-related quality of life in cancer patients receiving palliative care

**DOI:** 10.3389/fpsyg.2023.1166801

**Published:** 2023-05-26

**Authors:** Astrid Schnabel, Florian Lordick, Paula Oberth, Markus Neuschulz, Antje Lehmann-Laue, Anja Mehnert-Theuerkauf, Andreas Hinz

**Affiliations:** ^1^Department of Medicine (Oncology, Gastroenterology, Hepatology, Pulmonology), University of Leipzig Medical Center, Comprehensive Cancer Center Central Germany (CCCG), Leipzig, Germany; ^2^Department of Medical Psychology and Medical Sociology, University of Leipzig, Leipzig, Germany

**Keywords:** supportive care needs, palliative care, cancer, quality of life, importance, satisfaction

## Abstract

**Objective:**

Cancer patients receiving palliative care experience a variety of impairments in their quality of life (QoL), and have corresponding supportive care needs (SCNs). The aim of this study was to analyze the relationship between SCNs, satisfaction with QoL dimensions, and the perceived importance of these dimensions.

**Method:**

A sample of 152 cancer patients receiving palliative care were included in this cross-sectional study. Eight dimensions of QoL were defined and assessed concerning SCNs, satisfaction, and subjective importance using a new assessment instrument with five-point scales (range 1–5) for each dimension.

**Results:**

Among the eight specific domains examined, the greatest SCNs were observed for *absence of pain* (*M* = 3.18; SD = 1.29). The patients were least satisfied with their *physical functioning* (*M* = 2.60; SD = 0.84), and the dimension *social relationships* (*M* = 4.14; SD = 0.72) received the highest perceived importance ratings. The eight dimensions’ SCNs scores were significantly correlated with each other (*r* between 0.29 and 0.79); the lowest correlations were found for *social relationships*. The correlations between the satisfaction scores and the SCNs differed from dimension to dimension, with coefficients between −0.32 (*absence of pain*) and − 0.57 (*sleep quality*).

**Conclusion:**

The results show that detriments in QoL do not automatically indicate high levels of SCNs in those dimensions. Health care providers should consider both factors, QoL (as measured with QoL questionnaires) and subjectively expressed SCNs, to optimize their patients’ care regimens.

## Introduction

Cancer is a global health concern, as reflected by the 19.3 million new cases and 10 million deaths due to cancer in 2020, figures that are expected to almost double by 2040 (World Health Organization, 2020).

Quality of life (QoL) has gained increasing attention in oncological practice and research (Hinz et al., 2018; Firkins et al., 2020; Wasalski and Mehta, 2021). Particularly in the field of palliative treatment, maintaining a high level of QoL is a central aim of care (Hoomani Majdabadi et al., 2022). This means a shift from a disease-centered conceptualization to a more person-centered approach. This understanding recognizes that palliative care should be delivered based on need rather than prognosis (Radbruch et al., 2020). The heterogeneity of impairments in QoL among patients with advanced cancer requires systematic monitoring of QoL, and early identification and referral of high-risk patients to palliative care (Lee et al., 2022). Whereas symptom management is a core element of palliative care, providing patients with the skills they need to cope with and communicate about their life-threatening illness is also an important intervention of palliative care for patients with advanced cancer (Temel et al., 2017). Since palliative care should be an active holistic care of individuals with serious health-related suffering due to severe illness, (Radbruch et al., 2020), one of the key aspects of palliative care is its interdisciplinary operation, which allows the palliative care team to deliver multidimensional care addressing the complex supportive care needs of patients with advanced cancer (Higginson and Evans, 2010; Hui et al., 2018).

Almost all patients with advanced, incurable cancer experience some level of need for help across a variety of domains (Rainbird et al., 2009). Corresponding to the patient-centered principles of modern oncology, needs should reflect the wishes of the patient rather than clinical judgments or interpretations of health-related measures (Osse et al., 2005; Moghaddam et al., 2016). These wishes can include physical, mental, and social aspects as well as the dimension of spirituality/religiosity (Delgado-Guay et al., 2021).

High levels of SCNs in cancer patients are predicted by high levels of distress, anxiety, depression, and physical symptoms as well as low QoL (Hwang et al., 2004; Snyder et al., 2009; Lehmann et al., 2012; Au et al., 2013; Lam et al., 2014; Beernaert et al., 2016; Sodergren et al., 2019; Choi et al., 2020; Wang et al., 2021). However, more precise analyses of the relationship between QoL and SCNs are difficult because the instruments for measuring QoL and those for measuring SCNs have been developed largely independently from one another, resulting in inconsistently designed dimensions.

Frequently used instruments designed for measuring SCNs are: the Supportive Care Needs Survey questionnaire SCNS-SF34 (Boyes et al., 2009) (scales: health system and information needs, psychological needs, physical and daily living needs, patient care and support needs, and sexuality), the Cancer Needs Questionnaire (Cossich et al., 2004; Sharour, 2021) (scales: psychological, health information, physical and daily living, patient care and support, and interpersonal communication needs), and the Cancer Survivorship Unmet Needs tool CaSUN (Hodgkinson et al., 2007; Chung et al., 2019) (scales: information, comprehensive cancer care, existential survivorship, QoL, and relationships), see also (Rimmer et al., 2022). These scales do not clearly correspond to the scales incorporated in QoL assessment instruments such as the EORTC QLQ-C30 (Aaronson et al., 1993) or the SF-36 (Ware and Sherbourne, 1992).

It is plausible that patients require supportive care especially in those domains for which they experience detriments and which are perceived as being important to them. The subjective importance of dimensions of QoL is not considered in most of the QoL assessment instruments; it is implicitly assumed that all dimensions are of equal relevance for the patients. There are instruments that explicitly include the subjective importance of the dimensions of QoL, e.g., the Schedule for the Evaluation of Individual Quality of Life (SEIQoL) (Joyce et al., 2003) and the Patient Generated Index (PGI) (Martin et al., 2007). In these instruments, however, the patients are free to name dimensions they personally consider important, resulting in highly heterogeneous dimensions. Therefore, these instruments are not suitable for the analysis of the relationship between importance, satisfaction, and SCNs for specific dimensions.

Because it is not possible to precisely compare SCNs, QoL, and subjective importance for specific dimensions with the existing measurement instruments, we developed a new instrument in which the questions about SCNs, quality of life, and subjective importance each refer to the same dimensions. This new instrument is the first to enable direct comparisons of SCNs, QoL, and subjective importance for the same dimensions.

Need for support depends on age. Younger patients reported higher levels of psychosocial needs than older ones (Lehmann et al., 2012), whereas older age was associated with higher physical and daily living unmet needs (Lehmann et al., 2012; Konstantinidis et al., 2016). Females showed a higher wish for support than males did, with the exception of the *sexuality* domain, for which the males desired more support (McDowell et al., 2010; Lehmann et al., 2012; Sakamoto et al., 2017). However, other studies failed to detect significant sex differences in the SCNs (Liao et al., 2011; Gebresillassie et al., 2021). These results were obtained for several samples of cancer patients albeit without taking the specific needs of palliative patients into account. As such, it remains unknown to what degree these results are generalizable to palliative patients. Therefore, we also intend to investigate age and sex differences in SCNs in a sample of patients receiving palliative care.

The aims of this study were (a) to determine levels of SCNs, satisfaction, and importance for specifically designed components of QoL, (b) to investigate the mutual relationships between SCNs on the one hand, and the dimensions’ subjective importance to patients and their satisfaction with them on the other, and (c) to explore to what degree SCNs depend on sociodemographic and clinical factors.

## Methods

### Sample of cancer patients

The study participants were recruited in a German university hospital between November 2020 and May 2022. A total of 250 consecutive patients were asked to participate, 152 of whom (60.8%) agreed to take part in the study and complete the questionnaires. The sample consisted mainly of patients seeking outpatient palliative care counseling (137 patients). In addition, between January and May 2022, patients were recruited from an inpatient palliative care facility (15 patients). All patients with a palliative, incurable cancer diagnosis were eligible for the study. Exclusion criteria were insufficient command of the German language and the presence of severe cognitive impairment. The Ethics Committee of the University of Leipzig approved the study. Written informed consent was obtained from the participants after they were given a full explanation of the purpose and nature of the data collection and storage.

### Instruments

No instrument was available for the joint assessment of SCNs, QoL, and perceived importance of the dimensions. Therefore, we selected the following eight dimensions of health-related QoL based on the scales of relevant QoL questionnaires such as the EORTC QLQ-C30 (Aaronson et al., 1993) and the SF-36 (Ware and Sherbourne, 1992): *physical functioning, autonomy, emotional stability, cognitive functioning, social relationships, vitality, absence of pain,* and *sleep quality* for measuring SCNs, satisfaction, and subjective importance. In addition to the eight specific dimensions, the participants were also asked to assess their global health state in terms of SCNs, satisfaction, and subjective importance. The first five of the eight specific dimensions were adopted from the functioning scales of the QoL questionnaire EORTC QLQ-C30. *Vitality* was taken from the SF-36; this dimension can be considered the opposite of *fatigue,* which is also included in the EORTC QLQ-C30. Pain is also a component of the SF-36 and the EORTC QLQ-C30. In contrast to the SF-36, we preferred to use the term “*absence of pain”* instead of “*pain”* so that high scores indicate high levels of QoL for all of the scales including *pain*. *Sleep quality* is a component of the EORTC QLQ-C30 but not of the SF-36. We also included this dimension because of its special relevance for cancer patients (Otte et al., 2015; Hofmeister et al., 2020).

Each of these nine dimensions (eight specific dimensions and one general dimension) had to be evaluated from three perspectives: “To what degree do you wish support in (e.g., physical functioning)?” (SCNs), “How satisfied are you with your (e.g., physical functioning)?” (satisfaction), and “How important is (e.g., physical functioning) for you?” (importance).

For each question, there were five response options (coded from 1 to 5): “To what degree do you wish support …” (no support at all, …, a lot of support), “How satisfied are you with your …”: (very dissatisfied, …, very satisfied), and “How important is …”: (not important, …, very important).

In addition, the patients were asked to assess changes in their satisfaction with the dimensions and in the importance of those dimensions to them, also using five-point scales. The questions and possible responses read as follows: “How did your satisfaction (e.g., with physical functioning) change from the time before being diagnosed and now” (I became much less satisfied, …, much more satisfied), and “How did the importance (e.g., of physical functioning) to you change from the time before you were diagnosed and now” (it became much less important, …, much more important”). These responses were also coded from 1 to 5.

### Statistical analysis

The differences between the study group and the non-participants were statistically tested with *t*-tests (mean age) and chi^2^-tests (sex and tumor type). Descriptive statistics of the SCNs, satisfaction ratings, and importance ratings were performed. The relationships between these variables were calculated with Pearson correlations, and the effects of sex, age group, setting (inpatient or outpatient), and tumor type on SCNs were statistically tested with *t*-tests and one-way analyses of variance (ANOVAs). Effect sizes according to Cohen were calculated to indicate the magnitude of the group mean differences in relation to the standard deviations. All statistics were performed with the statistics program SPSS, version 27.

## Results

### Sample characteristics

A total of 152 patients (response rate: 60.8%) were willing to complete the questionnaire, 63 males and 89 females, with a mean age of 65.1 ± 12.2 years (range: 32–86 years). Table 1 presents further characteristics of the study participants. The group of the non-participants consisted of 98 patients, 38 males and 60 females, with a mean age of 60.7 (SD = 14.9) years and the following frequencies of tumor diagnosis groups: gastrointestinal tract (*n* = 38), female genital organs (*n* = 22), breast (*n* = 21), kidney, urinary tract, prostate (*n* = 5), and others (*n* = 12). Of the 98 non-participants, 39 died within the study period. The group of non-participants was significantly younger than the study group (*t* = 2.550, *p* = 0.011), but there were no statistically significant differences between the groups concerning sex distribution (chi^2^ = 0.177, *df* = 1, *p* = 0.674) and tumor localization (chi^2^ = 1.993, *df* = 4, *p* = 0.737).

**Table 1:** Sociodemographic and clinical characteristics of the sample of palliative cancer patients (*n* = 152).

	*n*	%
Sex		
Males	63	41.4
Females	89	58.6
Age group		
≤ 59 years	46	30.3
60–69 years	50	32.9
≥ 70 years	56	36.8
Marital status		
Single	16	10.5
Married	90	59.2
Divorced/living separately	24	15.8
Widowed	22	14.5
Education^a^		
Elementary school (8–9 years)	28	18.5
Junior high school (10 years)	62	41.1
High school/university (≥ 11 years)	60	39.7
No formal qualification	1	0.7
Tumor localization		
Gastrointestinal tract	59	38.8
Female genital organs	32	21.1
Breast	25	16.4
Kidney, urinary tract, prostate	11	7.2
Other	25	16.4
Time since diagnosis^a^		
< 1 year	44	29.1
1 year - < 2 years	26	17.2
≥ 2 years	81	53.6
Setting		
Outpatient	137	90.1
Inpatient	15	9.9
Treatment		
Surgery		
No	50	32.9
Yes	102	67.1
Radiotherapy		
No	71	46.7
Yes	81	53.3
Chemotherapy		
No	26	17.1
Yes	126	82.9
Hormone therapy^a^		
No	114	75.5
Yes	37	24.5

a Missing data not reported.

### Mean scores for SCNs, satisfaction, and importance

Table 2 presents the mean scores of the SCNs values as well as the means of satisfaction and importance ratings, together with the changes in satisfaction and importance, separately for the eight dimensions of QoL and the *global health* assessment.

**Table 2:** SCNs, satisfaction, and importance ratings (range: 1–5).

	Phys. funct.	Auto-nomy	Emot. stability	Cogn. funct.	Social relat.	Vitality	Absence of pain	Sleep quality	Global health
SCNs *M*(SD)	2.74	2.49	2.48	2.16	1.81	2.60	3.18	2.47	3.24	(1.28)	(1.26)	(1.08)	(1.15)	(1.07)	(1.11)	(1.29)	(1.30)	(1.16)
Satisfaction *M*(SD)	2.60	3.19	3.21	3.31	4.14	2.92	3.32	3.16	2.88	(0.84)	(1.01)	(0.85)	(0.85)	(0.77)	(0.79)	(0.93)	(1.04)	(0.85)
Importance *M*(SD)	3.58	3.91	3.86	3.77	4.14	3.68	3.99	3.94	3.95	(0.69)	(0.76)	(0.68)	(0.63)	(0.72)	(0.70)	(0.77)	(0.66)	(0.70)
Satisfaction change *M*(SD)	1.83	2.24	2.37	2.47	3.17	2.15	2.38	2.49	1.97	(0.86)	(0.86)	(0.84)	(0.81)	(0.85)	(0.81)	(0.89)	(0.92)	(0.92)
Importance change *M*(SD)	3.42	3.72	3.66	3.47	3.70	3.56	3.88	3.69	3.95	(1.02)	(0.90)	(0.80)	(0.79)	(0.87)	(0.84)	(0.95)	(0.88)	(0.93)

*M*, Mean; SD, standard deviation.

The highest levels of SCNs (scale range: 1–5) were found for the dimensions *global health* (*M* = 3.24) and *absence of pain* (*M* = 3.18), while the dimensions with the lowest scores were *cognitive functioning* (*M* = 2.16) and *social relationships* (M = 1.81). The means of the remaining dimensions ranged from 2.47 to 2.74.

Regarding satisfaction, the highest score was reached in the *social relationships* dimension (*M* = 4.14), while the patients were most dissatisfied with their *physical functioning* (*M* = 2.60) and their *global health* (*M* = 2.88).

The importance ratings showed that all dimensions were important to the patients with mean scores above the scale mean of 3. The highest importance was attributed to *social relationships* (*M* = 4.14).

Concerning changes in satisfaction and importance, all dimensions (with the exception of *social relationships*) showed a decrease in satisfaction since the time of diagnosis as indicated by a score below 3; the strongest decline was experienced for *physical functioning* (*M* = 1.83) and *global health* (*M* = 1.97). All dimensions gained in importance following the occurrence of the disease, with scores between 3.42 and 3.95 on a scale of 1–5 with 3 as the neutral point.

### Correlations between SCNs, satisfaction, and importance of QoL dimensions

Table 3, upper part, shows the correlations among the SCNs scores. All SCNs were positively correlated with each other; most correlations ranged between 0.40 and 0.60. The lowest correlations were found for *social relationships* and the other variables. The minimum correlation (*r* = 0.29) was that between *social relationships* and *global health*. High correlations were observed between *autonomy* and *physical functioning* (*r* = 0.79) and between *absence of pain* and *global health* (*r* = 0.69).

**Table 3:** Correlations among the SCNs dimensions (upper part), and correlations between SCNs and satisfaction and importance ratings (lower part).

	Phys. funct.	Auto-nomy	Emot. stability	Cogn. funct.	Social relat.	Vitality	Absence of pain	Sleep quality	Global health
Correlations within SCNs									
Physical functioning	–	0.79***	0.59***	0.65***	0.32***	0.60***	0.50***	0.49***	0.55***
Autonomy		–	0.64***	0.67***	0.51***	0.58***	0.56***	0.52***	0.54***
Emotional Stability			–	0.67***	0.51***	0.61***	0.46***	0.51***	0.47***
Cognitive functioning				–	0.59***	0.59***	0.46***	0.49***	0.43***
Social relationships					–	0.40***	0.36***	0.48***	0.29***
Vitality						–	0.59***	0.59***	0.53***
Absence of pain							–	0.60***	0.69***
Sleep quality								–	0.58***
Global health									–
Correlations between SCNs and other variables									
Satisfaction	−0.35***	−0.49***	−0.36***	−0.54***	−0.35***	−0.42***	−0.32***	−0.57***	−0.32***
Importance	0.25**	0.03	0.05	0.08	−0.29***	0.28***	0.29***	0.34***	0.31***
Satisfaction change	−0.16*	−0.43***	−0.17*	−0.48***	−0.10	−0.28***	−0.25**	−0.38***	−0.27***
Importance change	0.14	0.20*	0.13	0.28***	−0.02	0.07	0.26**	0.34***	0.24**

*p < 0.05; **p < 0.01; ***p < 0.001.

The lower part of Table 3 presents the correlations between SCNs and other variables (satisfaction, importance, change in satisfaction, and change in importance) for each of the dimensions. For example, SCNs in *physical functioning* and satisfaction with *physical functioning* correlated negatively with *r* = −0.35. All correlations between the SCNs and the corresponding satisfaction assessments were negative, meaning that if patients were dissatisfied with a dimension they tended to seek support. This relationship is most pronounced in *sleep quality* (*r* = −0.57).

Dimensions that are perceived as being important to the patients were generally associated with higher levels of SCNs; all correlations but one were positive (Table 3). That exception concerns *social relationships*. The negative correlation (*r* = −0.29) means that patients who perceive social relationships as being important do not tend to ask for support in this field.

Overall, the correlations between SCNs and the change scores (change in satisfaction and change in importance) showed similar patterns as the correlations with the dimensions satisfaction and importance (Table 3).

If the eight specific SCNs scores were considered items of a scale measuring need for support, the resulting Cronbach alpha coefficient was 0.90. If, instead of the SCNs scores, the satisfaction scores were considered a scale, the alpha coefficient was 0.82, and the importance scores resulted in an alpha coefficient of 0.84.

### Effects of sociodemographic and clinical factors on SCNs

Sex differences in the SCNs were small in magnitude (Table 4). No effect size exceeded the value of *d* = 0.13. A statistically significant age difference was found for *social relationships*: younger patients reported lower levels of SCNs in this dimension with an effect size of *d* = 0.34.

**Table 4:** SCNs broken down by sex, age group, setting, and tumor type.

	Phys. funct.	Auto-nomy	Emot. stability	Cogn. funct.	Social relat.	Vitality	Absence of pain	Sleep quality	Global health
Sex											
Males	*M*	2.83	2.42	2.48	2.19	1.76	2.56	3.21	2.45	3.21	
	(SD)	(1.30)	(1.25)	(1.04)	(1.10)	(0.88)	(1.08)	(1.34)	(1.34)	(1.19)	
Females	*M*	2.67	2.54	2.48	2.14	1.85	2.63	3.16	2.49	3.26	
	(SD)	(1.26)	(1.27)	(1.11)	(1.20)	(1.19)	(1.14)	(1.25)	(1.28)	(1.15)	
Effect size		−0.13	0.10	0.00	−0.04	0.09	0.06	−0.04	0.03	0.04	
*t*-test	*p*	0.477	0.583	0.966	0.787	0.627	0.742	0.828	0.867	0.778	
Age group											
≤ 66 years	*M*	2.70	2.32	2.41	2.18	1.63	2.65	3.25	2.54	3.26	
	(SD)	(1.30)	(1.32)	(1.11)	(1.26)	(0.98)	(1.18)	(1.30)	(1.38)	(1.11)	
≥ 67 years	*M*	2.78	2.65	2.54	2.14	1.99	2.55	3.12	2.41	3.22	
	(SD)	(1.26)	(1.19)	(1.05)	(1.05)	(1.12)	(1.05)	(1.29)	(1.22)	(1.22)	
Effect size		0.06	0.26	0.12	−0.03	0.34	−0.09	−0.10	−0.10	−0.03	
*t*-test	*p*	0.712	0.115	0.485	0.841	0.040	0.614	0.536	0.573	0.848	
Setting											
Outpatient	*M*	2.69	2.40	2.48	2.14	1.79	2.56	3.12	2.47	3.23	
	(SD)	(1.29)	(1.27)	(1.08)	(1.14)	(1.06)	(1.15)	(1.30)	(1.31)	(1.18)	
Inpatient	*M*	3.20	3.29	2.47	2.33	2.00	2.93	3.73	2.47	3.33	
	(SD)	(1.01)	(0.91)	(1.13)	(1.29)	(1.13)	0.70)	(1.03)	(1.19)	(1.05)	
Effect size		0.44	0.82	−0.01	0.16	0.19	0.40	0.52	0.00	0.09	
*t*-test	*p*	0.140	0.012	0.962	0.548	0.464	0.222	0.081	0.985	0.748	
Tumor type											
Gastrointestinal tract	*M*	2.66	2.32	2.38	1.97	1.81	2.47	3.17	2.48	3.22	
	(SD)	(1.29)	(1.29)	(1.06)	(1.08)	(1.02)	(1.13)	(1.39)	(1.40)	(1.23)	
Female genital organs	*M*	2.74	2.45	2.73	2.17	1.72	2.75	3.20	2.48	3.48	
	(SD)	(1.37)	(1.15)	(1.20)	(1.34)	(1.22)	(1.14)	(1.21)	(1.40)	(1.06)	
Breast	*M*	2.71	2.62	2.54	2.46	2.08	2.83	3.08	2.56	3.16	
	(SD)	(1.08)	(1.24)	(1.06)	(1.06)	(1.32)	(1.13)	(1.26)	(1.12)	(1.14)	
Kidney, urinary tract, prostate	*M*	2.82	2.10	2.30	2.18	1.40	2.18	2.82	2.10	2.73	
	(SD)	(1.40)	(1.29)	(1.25)	(1.40)	(0.52)	(1.17)	(1.54)	(1.20)	(1.35)	
Others	*M*	2.74	2.49	2.48	2.16	1.81	2.60	3.18	2.47	3.24	
	(SD)	(1.28)	(1.26)	(1.08)	(1.15)	(1.07)	(1.11)	(1.29)	(1.30)	(1.16)	
ANOVA	*p*	0.932	0.333	0.415	0.524	0.508	0.480	0.739	0.873	0.540	

*M*, mean; SD, standard deviation.

Inpatients had higher levels of SCNs than outpatients in six of the eight specific dimensions, especially in the dimensions *autonomy* (*d* = 0.82) and *absence of pain* (*d* = 0.52), but all these differences failed to be statistically significant with the exception of *autonomy*. There were also no statistically significant differences among the tumor types; the *p* values for all dimensions were far from being statistically significant.

## Discussion

The first objective of this study was to analyze the SCNs in cancer patients receiving palliative care, and to explore the relationship between these SCNs and satisfaction and importance ratings. Among the eight specific dimensions, *absence of pain* was given the highest SCNs ratings (*M* = 3.18), while the lowest degree of needs for support was found for *social relationships* (*M* = 1.81) and *cognitive functioning* (*M* = 2.16) Obviously, *absence of pain* is relevant to the patients, and they also furthermore believe that health care providers can treat this symptom effectively (e.g., prescribing appropriate medication). The low SCNs for *cognitive functioning* cannot be explained by low dissatisfaction or importance scores. It seems likely patients do not believe it is their physician’s job to improve *cognitive functioning.*

*Social relationships* play a very specific role in the context of SCNs, satisfaction, and importance. This dimension is of maximum importance to the patients (*M* = 4.14), as seen in another German study with palliative patients (Vogt et al., 2021), but the patients seem to be pleased with that dimension and do not see the necessity of receiving support from health care providers in that area. Presumably they consider *social relationships* as a domain for which they are responsible themselves. The special role of *social relationships* is also underlined by the fact that *social relationships* are the only dimension with an increase in satisfaction from the time of diagnosis (M > 3.0), while all other dimensions showed a decrease (*M* < 3.0) in satisfaction.

All correlations among the SCNs for the specific dimensions and global health were positive, with coefficients between 0.29 and 0.79. This is in line with a study with cancer patients (Azman et al., 2021) which found correlations among the dimensions of the SCNS-SF34 ranging from 0.28 to 0.57. The highest correlation in our study was found for the relationship between *physical functioning* and *autonomy*, indicating the role of physical capacity for an autonomous life and participation in social life. Once more, the dimension *social relationships* played a specific role among the eight dimensions, in this case, exhibiting the lowest correlations to the other dimensions. Though there was also a positive correlation between SCNs in *social relationships* and SCNs in *global health* (*r* = 0.29), this correlation was the lowest among the eight specific dimensions. The correlation between *absence of pain* and *global health* (*r* = 0.69) was the highest association between a single component and SCNs concerning *global health*. This underlines first and foremost that patients expect physicians to assist in reducing their pain.

From the positive correlations among the SCNs scores, it can be concluded that all SCNs have a certain variance in common, which is also reflected in the Cronbach alpha coefficient of the SCNs dimensions. This may have several reasons. First, it is possible that all SCNs depend, at least in part, on the overall health situation of the patients: Patients with more health problems generally seek more support in all dimensions. The second possible explanation is that seeking help is a kind of personality trait: Some people tend to admit their need for help or their wish for help in all dimensions, while other people prefer to manage problems without external help. The third possible reason is an acquiescence effect or yes-set effect. Acquiescence is the general tendency to give affirmative answers to questions, irrespective of the content (Rammstedt et al., 2017), which leads to an artificial positive correlation. Unfortunately, our study does not allow us to determine to what extent each of these three possible mechanisms contributes to the correlations among the SCNs ratings.

All correlations between the SCNs and the satisfaction ratings were negative (*r* between −0.32 and − 0.57). This means that those patients who are dissatisfied with a component of their QoL tend to seek more support. This general tendency of associations between SCNs and QoL has also been reported in previous studies (Lehmann et al., 2012; Choi et al., 2020; Jie et al., 2020). However, these studies used instruments with different dimensions for measuring SCNs and QoL; the dimensions of the SCNs (e.g., assessed with the SCN-SF-34) did not precisely match the dimensions of the QoL assessment instruments. In our study, it was possible to compare the dimensions separately with the unified assessment instrument. The strongest effect of the opposing relationship between SCNs and satisfaction was found for *sleep quality* (*r* = −0.57), which underlines not only the importance of sleep problems but also the perceived need for help. Though all correlations between SCNs and corresponding satisfaction ratings were negative, they were of only moderate size (*r* between −0.32 and − 0.57). QoL and perceived need for help should be considered two related but nevertheless different topics, and health care providers should not restrict their attention to only one of these aspects (Lehmann-Laue et al., 2019).

There were no significant sex differences in the SCNs in our study. In the literature on SCNs, female cancer patients sometimes reported more SCNs than male cancer patients did (Cossich et al., 2004; Wessels et al., 2010; Lehmann et al., 2012). The lack of sex differences in our study may be due to the restriction to patients with advanced cancer.

Concerning age differences, the results were not uniform. While older patients reported higher SCNs than younger patients in four of the eight specific dimensions, the relationship was reversed for the other four dimensions. Regarding the different dimensions investigated, there was only one significant age difference: Older patients perceived more SCNs in *social relationships* than younger ones (*d* = 0.34). Here one has to take into consideration that *social relationships* were the dimension with the lowest levels for SCNs in males and females as well. Younger palliative cancer patients seem to have social networks for which they do not need external help. Our data set was not sufficiently large to derive definite conclusions about sex and age differences in SCNs of palliative care patients. Further research is needed to obtain resilient data for this group of patients as well.

As was to be expected, patients in the hospital setting reported more SCNs than those in outpatient care. However, due to the low sample size of the inpatient setting, it is not possible to derive generalizable conclusions. The only significant difference was in the area of autonomy, which is unsurprising when doing a comparison between inpatient and outpatient settings. As the concept of “early palliative care” is implemented in our outpatient oncological services, lower SCNs in these patients could be discussed as a potentially beneficial effect of these services (Temel et al., 2017; Vanbutsele et al., 2018).

There were also no statistically significant differences in SCNs regarding tumor types. Nevertheless, in six of the eight specific dimensions, as well as in *global health*, the tumor group “kidney, urinary tract, and prostate” reported the lowest SCNs. This corresponds to the relatively low levels of depression and distress reported by prostate cancer patients (Hinz et al., 2019; Esser et al., 2020). Due to the relatively low sample sizes in our study this result cannot, however, be generalized.

This study was restricted to SCNs in patients. QoL and SCNs of family caregivers were not the subject of our research irrespective of its possible correlation with QoL and SCNs of patients [17] in the context of palliative medicine. SCNs of family caregivers are gaining increasing relevance (Chua et al., 2020; Wang et al., 2021; Kim et al., 2022; Lund et al., 2022). It should also be taken into account that patients’ SCNs as reported by their family caregivers may differ from patients’ own assessments, as well as those of their physicians (Nair et al., 2019), a fact that needs to be acknowledged when patients and physicians are setting treatment goals together (Hong et al., 2021).

What helps patients express their psychological and palliative care support needs? Access to informational materials should be offered at low thresholds, and symptoms/distress and needs should be systematically assessed and need-based counseling services actively offered. The treatment team should be appropriately sensitized, strengthened and repeatedly trained to proactively offer patients discussions on the topic of palliative care and end-of-life care, as well as to assess the need for palliative care and pave the way for further offers. Sufficient resources are to be made available for this on the part of the health care system. The support of the social discourse of understanding dying and death as part of life is also worth mentioning here.

Several limitations of this study should be mentioned. Most of the patients were recruited in the context of outpatient care. If the study had included more patients from inpatient settings, specialized palliative wards, or hospices, the burden would probably have been more severe, and the SCNs might weigh differently. The study was performed in the German health care system; it must be kept in mind that other ways of expressing SCNs could prevail in other cultures (Molassiotis et al., 2017; Wang et al., 2022). Ethnic and racial minorities as well as cancer patients with intellectual disabilities might have different SCNs than our cohort reported (Ní Shé et al., 2021; Mazor et al., 2022).

SCNs, QoL, and subjective importance of the domains were assessed with only one item for each of the eight dimensions. This limits the reliability of the assessments. However, single-item measures have been used in many investigations dealing with the importance of domains, even in assessments of broad constructs such as well-being (Conrad et al., 2017). All dimensions were assessed with single items in a uniform way, therefore, the degree of reliability will be similar for all these assessments, and comparisons between the eight dimensions are nevertheless fair.

Finally, in this study on SCNs, we did not ask whether the needs were met or unmet (Hodgkinson et al., 2007; Bağçivan et al., 2022), and we did not ask whether there were unexpressed needs (Heß et al., 2022). A detailed analysis of met and unmet needs in further studies would provide a better insight into the nature of the needs of palliative cancer patients.

Taken together, the study showed that it is possible to relate the fields of QoL and SCNs using a unified assessment instrument, and that the interplay of QoL and SCNs, related to specific dimensions, provides deeper insight into the information necessary for optimizing care of palliative cancer patients. It is common sense that in palliative medicine physicians should not only consider the clinical state of the patients but also their QoL. This study specifically adds the knowledge that assessing subjectively perceived SCNs provides additional relevant information necessary for tailoring individual treatment approaches in palliative care.

## Data availability statement

The raw data supporting the conclusions of this article will be made available by the authors, without undue reservation.

## Ethics statement

The studies involving human participants were reviewed and approved by Ethics Committee of the University of Leipzig. The patients/participants provided their written informed consent to participate in this study.

## Author contributions

AH, FL, and AM-T designed the study. AS, PO, AL-L, and MN prepared the material and collected data. AS and AH performed the statistical analyses. AS wrote the first draft. AS, AH, and AL-L wrote the final version. All authors contributed to the article and approved the final version.

## Funding

The study was funded by a grant from the German Cancer Aid (Grant no. 7011 3931). The authors acknowledge the support by the Open Access Publishing Fund of Leipzig University, supported by the German Research Foundation within the program Open Access Publication Funding.

## Conflict of interest

The authors declare that the research was conducted in the absence of any commercial or financial relationships that could be construed as a potential conflict of interest.

## Publisher’s note

All claims expressed in this article are solely those of the authors and do not necessarily represent those of their affiliated organizations, or those of the publisher, the editors and the reviewers. Any product that may be evaluated in this article, or claim that may be made by its manufacturer, is not guaranteed or endorsed by the publisher.
